# Predicting first falls among older adults with chronic conditions and polypharmacy using routinely collected health records

**DOI:** 10.3389/frai.2026.1852439

**Published:** 2026-09-04

**Authors:** Igor Larrañaga, Miguel Rujas, Itxaso Alayo, Irati Erreguerena, Marco Capo, Laura Lopez-Perez, Marta M. Mediavilla, María Dolores Martínez, Héctor Chuliá-Gil, Eleni I. Georga, Muhammad Salman Haleem, Leandro Pecchia, Dimitrios I. Fotiadis, Giuseppe Fico, Ane Fullaondo

**Affiliations:** 1 Biosistemak Institute for Health Systems Research, Bilbao, Spain; 2 Network for Research on Chronicity, Primary Care and Health Promotion (RICAPPS), Bilbao, Spain; 3 Research Unit, Debagoiena Integrated Healthcare Organisation, Osakidetza-Basque Health Service, Arrasate-Mondragón, Spain; 4 Life Supporting Technologies Research Group, Escuela Técnica Superior de Ingenieros de Telecomunicación, Universidad Politécnica de Madrid, Madrid, Spain; 5 Hospital Care Pharmacy, University Hospital Araba-Santiago, Araba Integrated Healthcare Organisation, Osakidetza-Basque Health Service, Vitoria-Gasteiz, Spain; 6 Mysphera, Paterna, Spain; 7 Unit of Medical Technology Intelligent Information Systems, Department of Materials Science and Engineering, University of Ioannina, Ioannina, Greece; 8 Biomedical Research Institute, Foundation for Research and Technology-Hellas, Ioannina, Greece; 9 School of Engineering, University of Warwick, Coventry, United Kingdom; 10 School of Electronic Engineering and Computer Science, Queen Mary University of London, London, United Kingdom; 11 Università Campus Bio-Medico di Roma, Roma, Italy

**Keywords:** artificial intelligence, chronic diseases, falls, first-fall prediction, machine learning, polypharmacy, predictive modelling

## Abstract

**Background:**

Falls represent a major clinical and financial challenge for healthcare systems. Accurately predicting first falls remains challenging, especially when using routinely collected data.

**Objective:**

Develop and evaluate a predictive model to identify elderly people at risk of first fall—defined as a fall after a 90-day fall-free period—in the Basque Country using routinely collected health records.

**Method:**

A retrospective study included patients aged ≥65 with at least two chronic conditions among heart failure, chronic obstructive pulmonary disease (COPD), and diabetes. Data on demographics, diagnoses, prescriptions and healthcare utilisation were obtained from Osakidetza-Basque Health Service databases. Patients were labelled as “fallers” if they fell during 2022–2023 after a 90-day fall-free period rather than a true first-ever fall. Predictive models—logistic regression (LR), random forest (RF), and extreme gradient boosting (XGB)—were trained using recursive feature elimination with cross-validation (RFECV). Shapley additive explanations (SHAP) enhanced model interpretability and explainability.

**Results:**

35,197 patients were included, with 10.6% experiencing a fall. All models achieved similar results, with an AUCROC score of 0.71, while precision remained low. Emergency room visits in the prior 3 months, presence of caregiver, and age consistently ranked the top predictors. The number of prescriptions and antidepressant use also emerged as relevant.

**Discussion:**

Models showed moderate predictive performance. Relying solely on routinely collected health records limited clinical applicability due to low precision. Future work should integrate diverse data sources—health records, real-time gait and balance metrics, environmental factors—to improve fall prediction and support clinical decision-making.

## 1 Introduction

In recent years, the population over 65 years and its life expectancy have increased (OECD, 2009; OECD, 2011). The growing longevity has led to a progressive rise in the prevalence of chronic diseases and associated polypharmacy (Allepuz Palau et al., 2015; Goodwin et al., 2012; Chang et al., 2020). As treatments become more complex and long-term (Brown and Bussell, 2011; Pérez et al., 2002; Núñez Montenegro et al., 2014), older adults face a higher risk of adverse events such as falls (Hanlon et al., 1997).

Falls represent a major public health issue and a significant clinical and financial challenge for healthcare systems (Chang et al., 2020). About 30% of people over 65 years and 50% of those over 80 experience a fall annually, with 75% of them likely to fall again (National Institute of Health and Care Excellence, 2013; Ministerio de Sanidad, Servicios Sociales e Igualdad, 2014). Falls are a leading cause of functional dependence in the elderly and the fifth leading cause of death among people over 65 years (Ministerio de Sanidad, Servicios Sociales e Igualdad, 2014). They are also associated with increased healthcare resource use (Campbell et al., 2004; INFAC, 2019; Steinman and Hanlon, 2010), accounting for 10% of emergency room visits and 6% of hospital admissions (Ministerio de Sanidad, Servicios Sociales e Igualdad, 2014), along with an associated increase in costs (INFAC, 2019). In addition, fear of falling is linked to negative psychological and behavioural effects, as well as reduced activity levels and lower quality of life (Scheffer et al., 2008; Schoene et al., 2019).

Given the negative health and economic impacts of falls, fall risk assessment and prevention have become a global healthcare priority (INFAC, 2019; Villafaina and Gavilán, 2011; Orueta Mendia et al., 2014). Some risk factors can be controlled (Lázaro del Nogal, 2009; Martínez et al., 2017; Balagué et al., 2015), such as the use of certain medications (Milos et al., 2014; de Vries et al., 2018; Fastbom and Schmidt, 2010), where timely interventions may contribute to fall prevention (Cameron et al., 2018; de Jong et al., 2013; Beers et al., 1991). In the Basque Country, 92% of accidents in people over 74 years in 2012 were due to falls, a third of which were preventable (INFAC, 2019).

Consequently, early detection of individuals at risk of falling is gaining interest among healthcare organisations (National Institute of Health and Care Excellence, 2013; Villafaina and Gavilán, 2011; Orueta Mendia et al., 2014). There is a pressing need for cost-effective prediction systems that can be applied in real-world settings (Rajagopalan et al., 2017). The secondary use of routinely collected data in clinical practise for this purpose could provide an effective solution (Lee et al., 2020), requiring no additional effort from already overburdened healthcare professionals (Duong and Vogel, 2023). Moreover, the digitalization of health records enables the integration of clinical and administrative data (Garrison et al., 2007; Menvielle et al., 2017; Noffsinger and Chin, 2000), facilitating the use of machine learning on large samples for the early detection of high-risk populations (Huang et al., 2021; Deschepper et al., 2019; Alowais et al., 2023). Combining electronic health records (EHR) with predictive models allows for the identification of key risk factors (Lee et al., 2020), providing healthcare professionals with interpretable models to support clinical decision-making (Orueta Mendia et al., 2014; Alowais et al., 2023).

Several studies have developed models to predict fall risk in elderly populations (Marier et al., 2016; Kuspinar et al., 2019; Lo et al., 2019; Millet et al., 2023; Lage et al., 2023; Lockhart et al., 2021; Noh et al., 2021; Dormosh et al., 2022)—using techniques such as logistic regression (Marier et al., 2016; Millet et al., 2023; Lage et al., 2023), decision trees (Kuspinar et al., 2019; Millet et al., 2023), random forest (Lo et al., 2019; Millet et al., 2023; Lockhart et al., 2021), and extreme gradient boosting (Noh et al., 2021)—, although few were based on routinely collected data (Marier et al., 2016; Dormosh et al., 2022; Dormosh et al., 2024). The multiple perspectives, methods, and predictors indicates that the field is still evolving (Deandrea et al., 2013). Most models include fall history among the analysed risk factors (Marier et al., 2016; Lo et al., 2019; Millet et al., 2023; Lage et al., 2023; Lockhart et al., 2021; Noh et al., 2021; Dormosh et al., 2022), which consistently emerges as the strongest predictor of a new fall (Pluijm et al., 2006; Stalenhoef et al., 2002), but limits their preventive value for first-time falls, an outcome that has been operationally defined in only one study as a fall occurring after a 90-day fall-free period (Kuspinar et al., 2019). Other models overlook prescriptions (Marier et al., 2016; Lo et al., 2019; Lockhart et al., 2021; Noh et al., 2021), despite the well-established role of certain drugs as modifiable fall risk factors (de Vries et al., 2018). Identifying individuals at risk of fall due to prescription-related issues is relevant for healthcare organisations to prevent adverse events and reduce costs (INFAC, 2019; Villafaina and Gavilán, 2011). Some studies also fail to use a sufficiently large sample size (Marier et al., 2016; Millet et al., 2023; Lage et al., 2023; Lockhart et al., 2021; Noh et al., 2021). Furthermore, limited reporting on model metrics (Marier et al., 2016; Kuspinar et al., 2019), especially in terms of precision (Marier et al., 2016; Kuspinar et al., 2019; Lage et al., 2023; Lockhart et al., 2021), highlights the need for further robust research in the field (Seaman et al., 2022).

In this context, the European GATEKEEPER project was launched to harness new technologies to improve care for an ageing population (de Batlle et al., 2023). In the Basque Country, an intervention for chronic disease and polypharmacy management was implemented, with concern for falls linked to medication interactions. This initiative offered a unique opportunity to develop a predictive model to shed light on fall risk factors and assess its performance using routinely collected health data.

The present study contributes to the field by developing and evaluating a supervised machine learning predictive model that uses routinely collected health records to predict first falls—operationally defined as a fall occurring after a 90-day fall-free period—and assess the role of polypharmacy in older adults with chronic conditions in the Basque Country, supporting early intervention and healthcare system sustainability.

## 2 Method

A retrospective study was conducted on the target population using anonymised data provided by Osakidetza, the Basque Health Service.

### 2.1 Target population

The target population was people registered in Osakidetza-Basque Health Service’s databases that on 1 January 2022 were aged 65 or older and had 2 or more chronic diseases between heart failure, chronic obstructive pulmonary disease (COPD) and diabetes. Comorbidity criteria were defined using international classification of diseases, ninth revision (ICD-9) and tenth revision (ICD-10) diagnostic codes. The ICD-9 codes were 398.91, 402.*1, 404.*1, 404.*3, 425.*, 428.* for heart failure, 490.*-496.* for COPD and 250.* for diabetes. The ICD-10 codes were I09.9, I11.0, I13.0, I13.2, I25.5, I42.*-I43.*, I50.* for heart failure, J40.*-J47.* for COPD and E10.*-E13.* for diabetes.

Patients who experienced a first fall between 1 January 2022 and 31 December 2023 were classified as positive cases or “fallers”, with the fall date set as the reference. Falls were defined as events occurring after a 90-day fall-free period, rather than as guaranteed first-ever falls. This 90-day window was chosen pragmatically to align the outcome definition with the 90-day predictor window available from routinely collected data, consistent with previous studies using administrative records (Kuspinar et al., 2019). For “non-fallers”, a random reference date within the same period was assigned. This case-reference design characterises the risk profile preceding falls that occur immediately after the 90-day observation window, rather than aiming to predict events within a predefined prospective time horizon.

### 2.2 Data source

**Figure 1:**
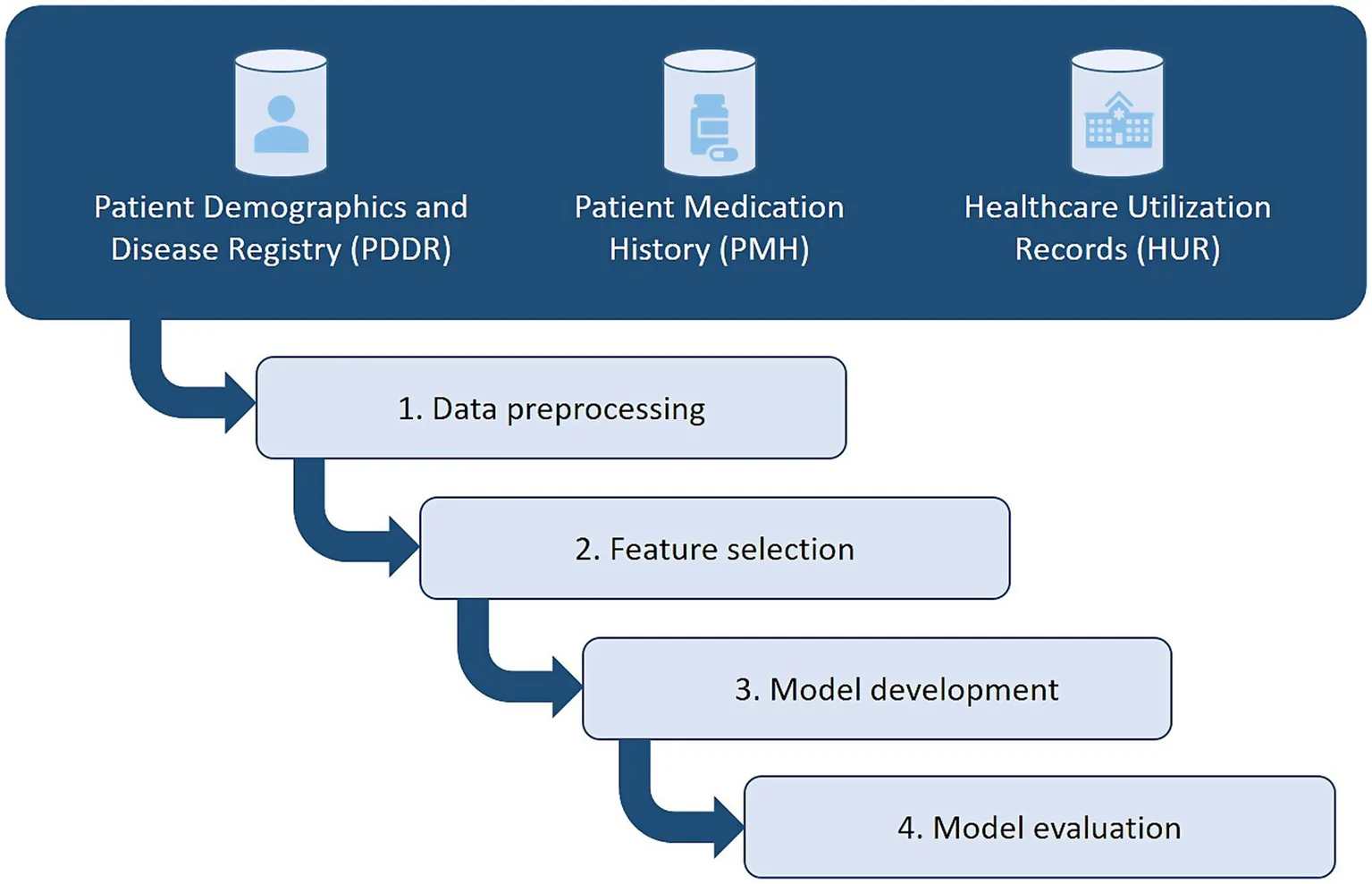
Overall methodology.

All data were sourced from Osakidetza-Basque Health Service’s anonymised corporative databases. The information was collected at the patient level and structured in three datasets (Figure 1). Patient demographics and disease registry (PDDR) included variables such as age, sex, socioeconomic status, deprivation index, Charlson index, ICD-9/10 diagnosis codes, fall history, and mortality. Patient medication history (PMH) recorded all prescriptions with start/end dates and anatomical therapeutic chemical (ATC) codes. Healthcare utilisation records (HUR) included, for primary care (PC), all contacts with medical and nursing staff, and for hospital care (HC), all contacts with outpatient services, emergency room, and hospitalisation. These data were obtained exclusively from the 90 days preceding the reference date. All records dated on or after the reference date, including the index fall encounter itself, were excluded to ensure that the models could not capture the fall event and to prevent any information leakage from the outcome into the predictive models.

### 2.3 Predictive model

Following the GATEKEEPER project methodological framework for developing artificial intelligence (AI) systems in healthcare, this study applied established supervised machine learning methods across four main phases depicted in Figure 1: data preprocessing, feature selection, model development, and model evaluation. It also adhered to the transparent reporting of a multivariable prediction model for individual prognosis or diagnosis plus artificial intelligence (TRIPOD+AI) statement (Collins et al., 2024), adopted here as a reporting standard to ensure comprehensive and transparent reporting of the machine learning methods used, with further details provided in the Supplementary Table S1. All analyses were performed using Python (version 3.9.7) and the scikit-learn library (Scikit-Learn, n.d.).

#### 2.3.1 Data preprocessing

First, a descriptive analysis of the sample was conducted. For categorical variables of two categories Fisher’s exact test was used, whereas the chi-square test was applied to the others; for continuous variables with a normal distribution, Student’s *t*-test was employed. Given the large sample size, the standardised mean difference (SMD) was also calculated using Cohen’s d for continuous variables and Cohen’s h for categorical variables to quantify between-group differences independently of statistical significance.

Subsequently, to integrate the three aforementioned datasets a comprehensive data engineering process was conducted under the supervision of healthcare professionals to ensure scientific and technical rigour. The PDDR dataset, which captures data at a single time point, served as the core, where the definitions of all diagnostic predictor variables and their corresponding ICD-10 codes are provided in the Supplementary Table S2. All modelled variables were complete by construction, and therefore no patient was excluded due to missing data; the full eligible cohort was analysed. The PMH dataset provided prescription data, aggregated at the 3rd ATC level—first four characters—as previously in the literature (Milos et al., 2014). For each patient, the number of active medications per subgroup on the reference date was recorded. The HUR dataset offered insights into healthcare resource usage over the 90 days before the reference date, aligning with first-fall definition. Pairwise correlations among variables were assessed considering all correlation values in absolute terms. For pairs with an absolute Pearson correlation coefficient greater than or equal to 0.8, the variable with the lower absolute correlation to the target was removed, thereby reducing multicollinearity and enhancing model interpretability.

The resulting final dataset preserved both clinical and temporal patterns while minimising data loss. It was split into training and test sets in an 80/20 ratio and standardised. Standardisation tools were trained on the training set and applied to the test set to prevent data leakage. Categorical variables with an ordinal structure were encoded using label encoding (LabelEncoder, n.d.), while numerical variables were scaled between 0 and 1 applying min-max normalisation based on the dataset’s minimum and maximum values (MinMaxScaler, n.d.).

#### 2.3.2 Feature selection

To reduce the number of variables and enhance model explainability, feature selection process was performed using recursive feature elimination with cross-validation (RFECV) (cv. = 5) on the training set (RFECV, n.d.; Misra and Yadav, 2020). This method automatically determines the optimal number of features by adjusting an estimator across different subgroups of variables, which are then evaluated using a scorer. Three different algorithms were employed in this process: logistic regression (LR) (Domínguez-Almendros et al., 2011), random forest (RF) (Breiman, 2001), and extreme gradient boosting (XGB) (Chen and Guestrin, 2016). These models were selected for their widespread use in clinical risk prediction and prior applications in fall risk estimation works (Millet et al., 2023; Sitompul et al., 2025). Deep learning models were excluded, as XGB is recognised as the standard choice for classification tasks involving tabular data (Shwartz-Ziv and Armon, 2022). Models were configured with default hyperparameters and class weighting to address dataset imbalance, leading to an optimised evaluation using the area under the curve precision-recall (AUCPR) metric (Davis and Goadrich, 2006; McDermott et al., 2025). This process provided three selected variable subsets, each used to train the corresponding fall prediction model, enabling the evaluation of how different feature selection strategies influence model performance and interpretability.

#### 2.3.3 Model development

The three algorithms—LR, RF, and XGB—were then trained on each of the three variable subsets identified during the feature selection phase. Before training, the hyperparameters were optimised using grid search with cross validation (cv. = 5), with AUCPR serving as the target metric (LaValle et al., 2004). The different hyperparameters and their explored values for each algorithm are detailed in the Supplementary Tables S3–S5. To mitigate the effects of class imbalance, higher weights were assigned to the minority class during model training. The weight for each class was computed based on its relative frequency in the training data, giving more importance to underrepresented cases. Weighting was preferred over resampling because it addresses class imbalance without generating synthetic cases, preserving the real data distribution and allowing the models to account for the minority (faller) class through the AUCPR metric. This choice is supported by evidence that resampling-based corrections can impair model calibration and over-estimate minority-class risk without improving discrimination (van den Goorbergh et al., 2022; Carriero et al., 2025).

#### 2.3.4 Model evaluation

Following the fine-tuning of hyperparameters, algorithms performance was assessed across all classes using several metrics (Scikit-Learn, n.d.): confusion matrix, area under the curve receiver operating characteristic (AUCROC) (Melo, 2013), AUCPR, accuracy, recall, and precision. Additionally, shapley additive explanations (SHAP) values were employed to quantify the contribution of each variable to the model’s predictions (Roth, 1988). SHAP values provide an interpretable measure of how much each variable increases or decreases the prediction for a given instance. This approach helps to identify the most influential variables, offering deeper insights into the model’s decision-making process and enhancing its interpretability and explainability.

## 3 Results

A total of 35,197 patients were included, with 10.6% experiencing falls (Table 1). No patient was excluded owing to missing or incomplete data. Women comprised 47% of the total, with a higher proportion in the fall group (51% vs. 46%). The mean age was 79.9 years, significantly higher among fallers (82.6 vs. 79.6). The Charlson comorbidity index was also higher in this group (6.6 vs. 5.4), as was chronic medication use (10.3 vs. 8.8 drugs). Caregivers were present for 36% of patients, with a higher presence in the fall group (56% vs. 34%). Given the large sample size, all comparisons reached statistical significance. In terms of effect size, caregiver presence showed the largest effect size (0.44), followed by chronic prescriptions (0.39), age (0.37), and the Charlson index (0.36), all representing small-to-moderate differences, whereas sex and socioeconomic status showed only small differences (≤ 0.12).

**Table 1:** Patient characteristics of the sample.

Variable		Total (*N* = 35,197)		No fall (*N* = 31,482)		Fall (*N* = 3,715)		*p*-value^a^	SMD^b^
		*n*	% (SE) or mean (SD)	*n*	% (SE) or mean (SD)	*n*	% (SE) or mean (SD)		
Sex	Women	16,473	46.8 (0.3)	14,591	46.3 (0.3)	1,882	50.7 (0.8)	<0.01	0.09
	Men	18,724	53.2 (0.3)	16,891	53.7 (0.3)	1,833	49.3 (0.8)		
Age	Mean		79.9 (8.1)		79.6 (8.1)		82.6 (7.5)	<0.01	0.37
	<80	17,433	49.5 (0.3)	16,155	51.3 (0.3)	1,278	34.4 (0.8)	<0.01	0.34
	≥ 80	17,764	50.5 (0.3)	15,327	48.7 (0.3)	2,437	65.6 (0.8)		
Socioeconomic status	Low	12,948	36.8 (0.3)	11,775	37.4 (0.3)	1,173	31.6 (0.8)	<0.01	0.12
	Medium	15,313	43.5 (0.3)	13,559	43.1 (0.3)	1,754	47.2 (0.8)		
	High	6,936	19.7 (0.2)	6,148	19.5 (0.2)	788	21.2 (0.7)		
Charlson index	Mean		5.5 (3.3)		5.4 (3.3)		6.6 (3.7)	<0.01	0.36
	0	11	0.0 (0.0)	9	0.0 (0.0)	2	0.1 (0.0)	<0.01	0.30
	1–2	5,979	17.0 (0.2)	5,623	17.9 (0.2)	356	9.6 (0.5)		
	3–4	10,668	30.3 (0.2)	9,749	31.0 (0.3)	919	24.7 (0.7)		
	≥ 5	18,539	52.7 (0.3)	16,101	51.1 (0.3)	2,438	65.6 (0.8)		
Chronic prescription	Mean		9.0 (4.0)		8.8 (3.9)		10.3 (3.7)	<0.01	0.39
	0–9	20,339	57.8 (0.3)	18,685	59.4 (0.3)	1,654	44.5 (0.8)	<0.01	0.30
	≥10	14,858	42.2 (0.3)	12,797	40.6 (0.3)	2,061	55.5 (0.8)		
Caregiver	No	22,492	63.9 (0.3)	20,837	66.2 (0.3)	1,655	44.5 (0.3)	<0.01	0.44
	Yes	12,705	36.1 (0.3)	10,645	33.8 (0.3)	2,060	55.5 (0.4)		

a Calculated using Fisher’s exact test or chi square test for categorical variables and Student’s *t*-test for continuous variables.

b Calculated using Cohen's d for continuous variables and Cohen's h for categorical variables.

SMD, standardised mean differences.

The dataset included 231 variables, with 77% from PMH, 21% from PDDR, and 2% from HUR, with a detailed breakdown by source provided in the Supplementary Table S6. The application of RFECV with the three estimators produced subsets of 183, 209, and 164 variables from LR, RF, and XGB, respectively. RFECV retained the feature subset maximising cross-validated AUCPR. The comparatively large subset reflects many low-magnitude but non-redundant signals, particularly in the medication (PMH) block. Details of these subsets and estimators are in the Supplementary Table S7. The outcomes of hyperparameter optimisation for the three models across the different database subsets are also presented in the Supplementary Table S8.

**Figure 2:**
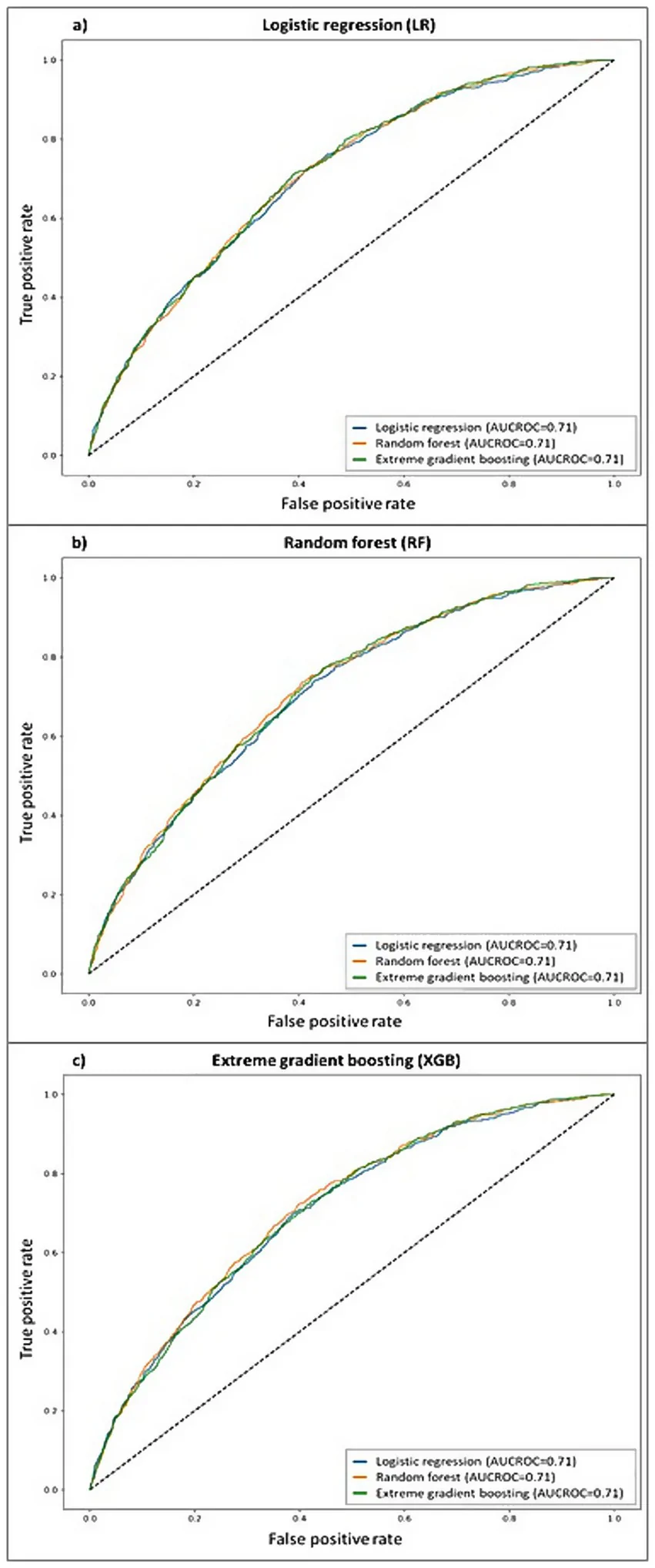
Receiver operating characteristic curves by feature selection and classification algorithm, respectively. AUCROC, area under the curve receiver operating characteristic.

**Figure 3:**
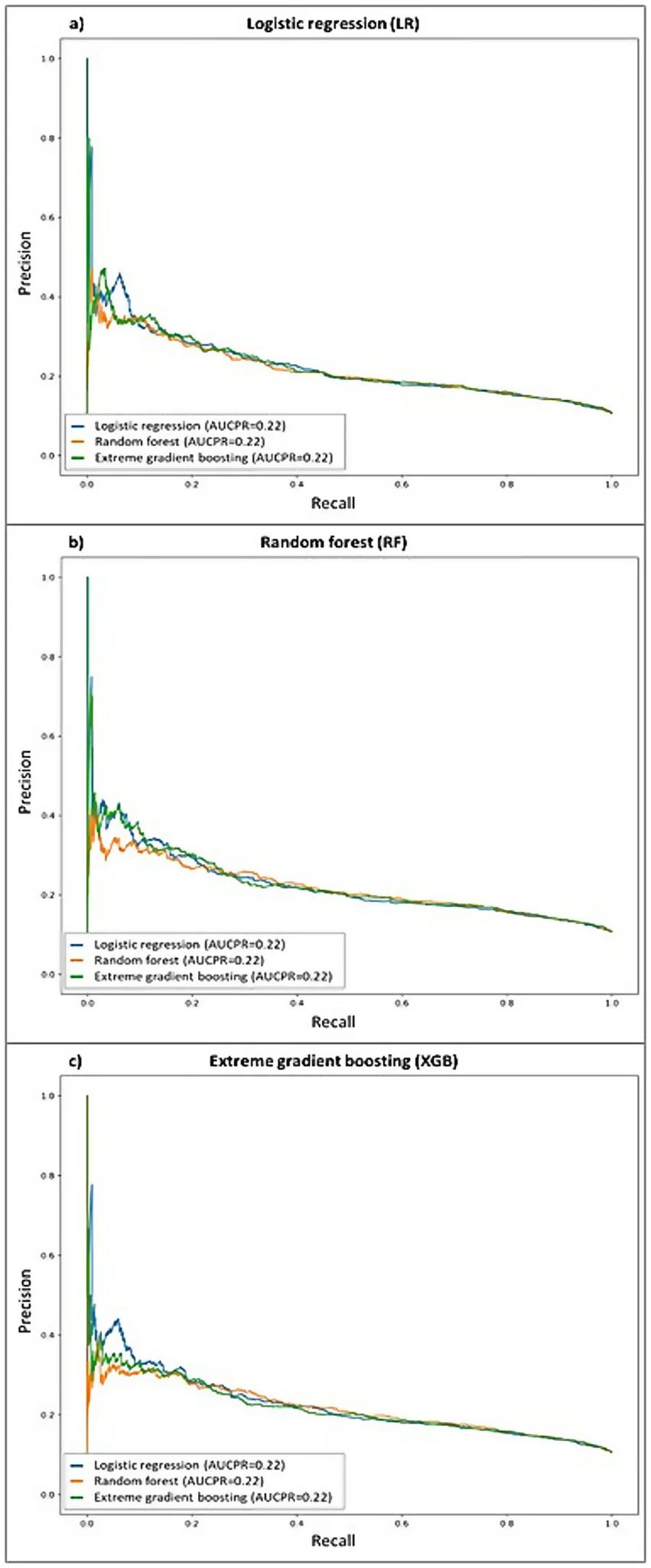
Precision-recall curves by feature selection and classification algorithm, respectively. AUCPR, area under the curve precision-recall.

The evaluation results of the algorithms are summarised in Figures 2, 3, with a more detailed version available in the Supplementary Table S9. All models achieved similar performance, reaching an AUCROC value of 0.71 and an AUCPR value of 0.22. For recall, the XGB model with LR-selected features reached the highest value of 0.62, whereas precision peaked at 0.41 for the RF model with RF-selected features. Given the pronounced class imbalance, accuracy is not emphasised in interpreting these results.

The SHAP values for the 10 most influential variables in each model are illustrated in Table 2, while a detailed analysis is provided in the Supplementary Figures S1–S3. The colour intensity in the table reflects the influence of the variable, with darker shades indicating stronger influence and lighter shades indicating weaker influence. Emergency room visits in the past 3 months, caregiver presence, and age were consistently the most impactful variables across all models, followed by hospitalisations and persistent atrial fibrillation. Among drugs, antidepressants (N06A) had the highest impact, followed by anxiolytics (N05B) and high-ceiling diuretics (C03C), with the number of prescriptions also standing out as noteworthy.

**Table 2:** Shapley additive explanations (SHAP) values of the 10 most influential features of each model.

Feature	Model								
	Selec.: LR	Selec.: LR	Selec.: LR	Selec.: RF	Selec.: RF	Selec.: RF	Selec.: XGB	Selec.: XGB	Selec.: XGB
	Class.: LR	Class.: RF	Class.: XGB	Class.: LR	Class.: RF	Class.: XGB	Class.: LR	Class.: RF	Class.: XGB
Number of ED visits in past 3 months	0.146	0.033	0.253	0.133	0.037	0.239	0.145	0.039	0.256
Caregiver	0.203	0.033	0.234	0.201	0.034	0.210	0.200	0.037	0.234
Age	0.241	0.029	0.232	0.222	0.031	0.196	0.238	0.033	0.224
Number of hospitalisations in past 3 months	0.070	0.022	0.121	0.067	0.024	0.123	0.070	0.023	0.122
Persistent atrial fibrillation	0.123	0.017	0.096	0.124	0.015	0.104	0.120	0.016	0.092
Number of PC nurse visits in past 3 months	–	0.021	0.112	0.047	0.019	0.112	0.054	0.021	0.104
Sex	0.116	–	0.100	0.105	–	0.081	0.110	–	0.085
Charlson points	–	0.019	0.075	–	0.014	0.068	–	0.016	0.069
Mild liver disease	0.081	–	–	0.083	–	–	0.081	–	–
Antidepressants (N06A)	0.059	–	0.081	0.051	–	0.070	0.056	–	0.082
Number of prescriptions	–	0.017	0.072	–	0.013	0.047	–	0.014	0.075
Diabetes mellitus with chronic complications	0.062	–	–	0.076	–	–	0.065	–	–
Number of outpatient visits in past 3 months	–	0.016	–	–	0.012	–	–	0.012	–
Number of PC doctor visits in past 3 months	–	0.015	–	–	–	–	–	0.012	–
Anxiolytics (N05B)	0.056	–	–	–	–	–	–	–	–
High-ceiling diuretics (C03C)	–	–	–	–	0.012	–	–	–	–

Selec., selection algorithm; Class., classification algorithm; LR, logistic regression; RF, random forest; XGB, extreme gradient boosting. Darker shades: stronger influence. Lighter shades: weaker influence.

## 4 Discussion

Our findings revealed a moderate performance for first-fall prediction models—that is, models predicting falls after a 90-day fall-free period—targeting elderly individuals with chronic diseases and polypharmacy. While the predictive variables differed slightly across the models developed (Table 2), all aligned in identifying the same three variables as the strongest contributors to the model’s fall predictions: age, presence of a caregiver, and emergency room visits in the previous 3 months. Throughout the manuscript, these SHAP-based contributions should be interpreted as model-specific explanatory measures and not as evidence of causal or mechanistic effects on fall risk. Age is a well-established risk factor, as older adults tend to experience a decline in balance, strength, and mobility (Millet et al., 2023; Noh et al., 2021). The presence of a caregiver should be interpreted with caution, as it most likely acts as a proxy for underlying frailty, disability, or dependence, as well as increased healthcare utilisation and documentation, reflecting a greater need for assistance with activities of daily living rather than representing a direct risk factor (Rubenstein, 2006). Similarly, the number of recent ER visits may signal underlying health issues. All three of these factors are associated with the model’s fall predictions, but when combined, they suggest a specific patient profile: a dependent older individual who, due to residual effects or ongoing health issues, faces an elevated risk of falling. As healthcare utilisation was measured in the 90 days before the fall, the patient profile identified by the models may partly reflect clinical deterioration preceding the fall rather than stable risk factors alone. However, this does not diminish the clinical relevance of these variables, as such deterioration represents a period of increased vulnerability during which the risk of falling is likely to be highest. Identifying this high-risk state may still provide a valuable opportunity for timely preventive interventions before the fall occurs, thereby averting its serious clinical and economic consequences. This suggests that the models may be better suited to identifying periods of acute, short-term fall risk—that is, transient states of increased vulnerability rather than directly modifiable causes of falls—rather than capturing long-term susceptibility to falling.

When analysing drug effect, the number of prescriptions emerges as a relevant factor, underscoring that the consequences of polypharmacy on patient safety are significant, with risks increasing for each additional medication (Gnjidic et al., 2012). In particular, antidepressants (N06A) ranked among the top ten factors with the greatest contribution to the model’s fall prediction (Milos et al., 2014; Fastbom and Schmidt, 2010). Antidepressants can cause dizziness, sedation, and orthostatic hypotension, or interact with other drugs, especially in older adults, leading to cognitive impairment and drowsiness. These effects may compromise balance and coordination, increasing fall risk. High-ceiling diuretics (C03C) and anxiolytics (N05B) were also identified among the most relevant factors, in line with previous studies on medication-related falls (Supplementary material) (Milos et al., 2014; Fastbom and Schmidt, 2010). Nevertheless, moderate performance of the models suggests that not all relevant predictor variables were considered in this study, and that additional factors influencing the likelihood of a fall may exist but were not captured by the models.

In terms of performance, all analysed models showed similar results, with an AUCROC score of 0.71 in line with previous studies (Lo et al., 2019; Millet et al., 2023; Lage et al., 2023; Lockhart et al., 2021; Noh et al., 2021; Dormosh et al., 2022). However, a key requirement for reliable fall prediction is balancing recall and precision. Recall indicates model’s ability to identify true falls, with the highest recall of 0.62 achieved by the XGB model using LR-selected features. Precision reflects the proportion of fall predictions that correspond to true falls. High precision is critical for maintaining a manageable workload when reviewing at-risk patients and for minimising the costs associated with false positives. In this case, the precision value dropped significantly to 0.18 as in other studies (Lo et al., 2019; Noh et al., 2021; Dormosh et al., 2022), where the highest observed value is 0.41. Consequently, the high number of false positives hinders the practical implementation of the model in clinical practise settings, requiring substantial effort to review and validate predicted cases. At the observed prevalence of 10.6%, of every 1,000 patients screened approximately 106 would experience a fall and 894 would not. Given the precision achieved across models (0.18 to 0.41), this would translate into between roughly 259 and 589 patients flagged as at risk, of whom between approximately 153 and 483 would be false positives. Rather than reducing workload, this would increase the burden on healthcare professionals and compromise the cost-effectiveness of the models (Duong and Vogel, 2023). This highlights that relying solely on routinely collected health records is lacking when it comes to developing a robust and practically implementable fall prediction model, a challenge that could apply to any healthcare system that systematically collects similar information. Furthermore, it should also be considered that these models were developed within Osakidetza—a publicly funded, vertically integrated single-payer healthcare system with a mature, shared electronic health record—and consequently, their transportability is likely to be greatest in settings with similarly integrated records and comparable coding practises and data structures, whereas predictor availability and model performance may differ in more fragmented or insurance-based healthcare systems.

At this stage, it is important to recognise that fall prediction and prevention are complex and multifactorial challenges (Bargiotas et al., 2023). Intrinsic and extrinsic factors, including physiological, psychological, behavioural, and environmental aspects, interact to influence fall risk (Rajagopalan et al., 2017). Consequently, comprehensively managing all these factors remains highly challenging to date, as does gathering related relevant information to improve predictive model performance (Bargiotas et al., 2023). The absence of frailty measures, cognitive screening, comprehensive assessments of mobility and physical function, and environmental hazard data likely limited the models’ ability to capture key mechanisms underlying falls. Consequently, the models relied on indirect, encounter-based proxies, which may partly explain their moderate discrimination and limited precision.

On one hand, relevant intrinsic data on gait and balance were unavailable. Although diagnoses of gait disorders, visual impairment, and/or vestibular dysfunction were available and included as predictors, they were binary indicators of prior diagnostic history rather than direct functional measures, likely constraining the discriminative and predictive performance achievable using routinely collected data alone. Technologies such as wearables and monitoring devices within the context of mobile health (mHealth) offer promising solution for gathering this information (Rajagopalan et al., 2017; Konara Mudiyanselage et al., 2025; Casilari et al., 2015), providing real-time insights into patients’ mobility patterns, balance stability, and overall physical activity levels (González-Castro et al., 2025). However, to date, the studies in the literature have only reported moderate performance in fall prediction models using these technologies (Konara Mudiyanselage et al., 2025). This suggests that integrating mHealth data with routinely collected health records may be key to making gait and balance information readily accessible and to enhancing model performance. Additionally, user acceptance and involvement in the monitoring process are crucial, as older adults often exhibit low engagement with wearable-based systems (Lage et al., 2023; Muñoz Esquivel et al., 2023). As a more immediately implementable intermediate step, functional assessments already collected in routine primary care—such as frailty measures, mobility assessments, and/or standardised physical performance tests—could, where available, enrich routinely collected records at lower technological and adoption barriers than wearable-based systems.

On the other hand, another major gap lies in the absence of environmental data, such as geographic location, home layout, and other extrinsic factors affecting patients. While many predictive models are developed in controlled environments, their performance often differs substantially when applied to real-world settings (Rajagopalan et al., 2017). In this study, the inability to capture contextual data—due to technological constraints such as limited access to new technologies or lack of integration with EHRs—restricted the understanding of patient mobility and living conditions, crucial for assessing external factors that may influence their health and well-being (Rajagopalan et al., 2017; Sczuka et al., 2023). Therefore, promoting the use and interoperability of data collected through technologies such as context-aware systems, monitoring devices, and ambient sensors is essential for identifying external fall risk factors, enhancing predictive models, and supporting targeted interventions for at-risk patients (Bargiotas et al., 2023; Job et al., 2020). Progress in this regard may also help overcome barriers to the adoption and widespread implementation of fall prevention systems in healthcare (Lee et al., 2020; Bargiotas et al., 2023; Ramadan et al., 2024).

Lastly, it is important to acknowledge that some falls occur purely by chance and are essentially unavoidable despite precautions, due to unpredictable factors like lapses in attention or sudden environmental changes. While prevention can reduce fall risks, not all falls can be prevented. However, given the high frequency and costs associated, even minor improvements in identifying high-risk individuals could result in significant cost savings.

Future work should prioritise user-centric longitudinal studies conducted in real-life conditions. This will require adaptable data-collection and fusion methods that leverage the strengths of each data source while ensuring compliance with patient-confidentiality regulations. It will also demand close interdisciplinary collaboration between IT and healthcare professionals to develop comprehensive fall prediction and prevention systems that support healthcare sustainability.

### 4.1 Limitations

The main limitation of this study lies in the inherent constraints of routinely collected data, which do not ensure complete and up-to-date information for every patient. While resource utilisation data are typically reliable due to consistent recording of service use, test and measurement data are only gathered when required for managing the patient’s condition—often during in-person consultations—so such information may not be available or up to date for all patients. Moreover, some measures, like the Barthel index and certain laboratory tests, have limited validity period and require frequent updates to remain accurate.

There is also the possibility that falls may be miscoded or not recorded at all, especially in cases where the fall did not result in injury or require medical attention (Hoffman et al., 2018). Information loss may be more substantial when integrating data from nursing homes and the private sector, due to shortcomings in data sharing (Vest and Kash, 2016). Consequently, this may have contributed to an underestimation of the total number of falls, with some true fallers likely misclassified as non-fallers, leading to conservative performance estimates.

Another limitation concerns the definition of first falls, based on a 90-day recall period. Defining falls as events occurring after a 90-day fall-free period is a pragmatic operational approach, and the authors acknowledge that it does not guarantee a true first-ever fall. While the timing and criteria used to define a first fall may be open to discussion, focusing on patients without a recent fall record remains valuable, as it enables predictive models to identify factors associated with falls and supports targeted preventive interventions. The way the first fall was defined in this study was intended to align the outcome definition with the predictor window available from routinely collected data, providing a practical basis for analysing factors associated with falls. The sensitivity of the findings to alternative washout periods was not assessed and remains an important area for future research, while a predefined prospective follow-up period also warrants further investigation.

A further limitation is the absence of external and temporal validation, as all models were developed and evaluated within a single healthcare system using an internal 80/20 split. Moreover, relying on a single random train–test split may produce performance estimates that are more optimistic and less robust than those obtained through repeated cross-validation or evaluation in independent datasets, further highlighting the need for external and temporal validation. However, given the moderate predictive performance, limited precision, and high false-positive burden, external validation is not warranted at this stage. The priority is first to determine whether routinely collected data, complemented by additional sources (e.g., balance and gait, environmental, and lifestyle information), can achieve clinically useful predictive performance. For the same reason, a formal calibration assessment and a decision-curve (net-benefit) analysis were not performed, as they would not alter this conclusion. As the models’ discrimination and precision are insufficient to support clinical use, a formal calibration assessment becomes informative only once a model achieves adequate discriminative performance, at which point both analyses would constitute relevant steps in future work. Given the models’ insufficient discrimination and precision for clinical use, evaluating calibration would have provided limited additional value, as a formal calibration assessment becomes informative only once a model achieves adequate discriminative performance, at which point both calibration assessment and decision-curve analysis would constitute relevant steps in future work.

## 5 Conclusion

Relying solely on routinely collected health records makes it challenging to develop a robust and implementable fall prediction model for clinical practise. Given the multifactorial nature of falls, a comprehensive interdisciplinary approach is essential. Future models should incorporate up-to-date patient data and integrate diverse sources beyond medical records, such as real-time balance and gait, environmental, and lifestyle information. On the basis of routinely collected records alone, the performance achieved is insufficient to support clinical use, and authors do not propose these models for implementation or external validation at this stage. Only if future models, enriched with such additional data sources, achieve adequate predictive performance would external validation and, potentially, clinical deployment become justified, with the aim of helping healthcare providers identify high-risk individuals, improving quality of life and reducing fall-related healthcare costs.

## Data Availability

The datasets presented in this article are not readily available because given the potentially sensitive nature of the data, the Ethics Committees of the participating healthcare provider organisations did not authorise public access. Dataset access requests should be made through the official channels of each organisation. Requests to access these datasets should be directed to igor.larranagauribeetxebarria@bio-sistemak.eus.

## Group member of GATEKEEPER Consortium

Claudio Caimi, Christian Tamporale, and Chiara Bonferini from Hewlett-Packard Italiana, Milan, Italy; Paolo Zampognaro and Federica Saca from Engineering Ingegneria Informatica S.p.A, Roma, Italy; Ioanna Drympeta and Konstantinos Votis from the Information Technologies Institute, Centre for Research and Technology Hellas, Thessaloniki, Greece; Frans Folkvord from PredictBy, Barcelona, Spain, and from Tilburg School of Humanities and Digital Sciences, Tilburg, Netherlands; Sergio Guillen and Juan-Carlos Naranjo from Mysphera S.L., Paterna, Spain; and Paula Curras, Jorge Posada, and German Gutierrez from Innova & European Projects Office, Integrated Health Solutions, Medtronic Ibérica S.A., Madrid, Spain.

## Glossary

AI: artificial intelligence
ATC: anatomical therapeutic chemical
AUCPR: area under the curve precision-recall
AUCROC: area under the curve receiver operating characteristic
COPD: chronic obstructive pulmonary disease
EHR: electronic health records
HC: hospital care
HUR: healthcare utilisation records
ICD-10: international classification of diseases, tenth revision
ICD-9: international classification of diseases, ninth revision
LR: logistic regression
mHealth: mobile health
PC: primary care
PCM: patients’ clinical measurements
PDDR: patient demographics and disease registry
PMH: patient medication history
RF: random forest
RFECV: recursive feature elimination with cross-validation
SHAP: shapley additive explanations
SMD: standardised mean difference
TRIPOD+AI: transparent reporting of a multivariable prediction model for individual prognosis or diagnosis plus artificial intelligence
XGB: extreme gradient boosting
