# Supplementary material for: Predicting first falls among older adults with chronic conditions and polypharmacy using routinely collected health records
Source: Front Artif Intell. 2026 Sep 4;9:1852439. doi: 10.3389/frai.2026.1852439 (PMC13586431; doi:10.3389/frai.2026.1852439)

Title: Predicting first falls among older adults with chronic conditions and polypharmacy using routinely collected health records. Supplementary material.

Table SM1 - TRIPOD AI statement for the predictive model.

TRIPOD+AI item	Manuscript section
1 (Title) & 2 (Abstract)	First page
3a-c (Background) & 4 (Objectives)	Introduction
5a-b (Data) & 6a-c (Participants)	Method: Target population and Data source
7 (Data preparation)	Method: Predictive model: Data preprocessing
8a-c (Outcome)	Method: Target population
9a-c (Predictors)	Method: Data source
10 (Sample size)	Results (1st Paragraph)
11 (Missing data)	Method: Predictive model: Data preprocessing
12a-g (Analytical methods)	Method: Predictive model
13 (Class imbalance) & 14 (Fairness)	Method: Predictive model: Feature selection
15 (Model output)	Method: Predictive model: Model evaluation
16 (Training versus evaluation)	Method: Predictive model: Data preprocessing and Model development
17 (Ethical approval)	Ethics approval
18a-f (Open science items: funding, conflicts of interest, protocol, registration, data sharing, code sharing)	Funding and Conflict of interest statements. Protocol not available, Study not registered and Proprietary Code and Data
19 (Patient and public involvement)	NA
20a-c (Participants)	Results (1st Paragraph)
21 (Model development) & 22 (Model specification)	Results and Supplementary material (Proprietary code)
23a-b (Model performance)	Results and Supplementary material
24 (Model updating)	Results and Supplementary material
25 (Interpretation) & 26 (Limitations)	Discussion
27a-c (Model usability)	NA

Table SM2 - Diagnostic variables and corresponding ICD-10 codes used in the study.

Variable	Diagnoses	ICD-10
charlson_01	Myocardial infarction	I21.*, I22.*, I25.2
charlson_02	Heart failure	I09.9, I.11.0, I13.0, I13.2, I25.5, I42.*-I43.*, I50.*
charlson_03	Peripheral vascular disease	I70.*, I71.*, I73.1, I73.8, I73.9, I77.1, I79.0, I79.2, K55.1, K55.8, K55.9, Z95.8, Z95.9
charlson_04	Cerebrovascular disease	G45.*, G46.*, H34.0, I60.*-I69.*
charlson_05	Dementia	F00.*-F03.*, F05.1, G30.*, G31.1
charlson_06	Copd	J40.*-J47.*
charlson_07	Connective tissue disease	M05.*, M06.*, M31.5, M32.*-M34.*, M35.1, M35.3, M36.0
charlson_08	Peptic ulcer disease	K25.*-K28.*
charlson_09	Benign hepatic disease	B18.*, K70.0-K70.3, K70.9, K71.3-K71.5, K71.7, K73.*, K74.*, K76.0, K76.2-K76.4, K76.8, K76.9, Z94.4
charlson_10	Diabetes mellitus without chronic complication	E10.0, E10.1, E10.6, E10.8, E10.9, E11.0, E11.1, E11.6, E11.8, E11.9, E12.0, E12.1, E12.6, E12.8, E12.9, E13.0, E13.1, E13.6, E13.8, E13.9, E14.0, E14.1, E14.6, E14.8, E14.9
charlson_11	Diabetes mellitus with chronic complication	E10.2-E10.5, E10.7, E11.2-E11.5, E11.7, E12.2-E12.5, E12.7, E13.2-E13.5, E13.7, E14.2-E14.5, E14.7
charlson_12	Hemiplegia or paraplegia	G04.1, G11.4, G80.1, G80.2, G81.*, G82.*, G83.0-G83.4, G83.9
charlson_13	Moderate or severe renal failure	I12.0, I13.1, N03.2-N03.7, N05.2-N05.7, N18.*, N19.*, N25.0, Z49.0-Z49.2, Z94.0, Z99.2
charlson_14	Cancer	C00.*-C26.*, C30.*-C34.*, C37.*-C41.*, C43.*, C45.*-C58.*, C60.*-C76.*, C81.*-C85.*, C88.*, C90.*-C97.*
charlson_15	Moderate or severe hepatic disease	I85.0, I85.9, I86.4, I98.2, K70.4, K71.1, K72.1, K72.9, K76.5, K76.6, K76.7
charlson_16	Metastasis	C77.*-C80.*
charlson_17	Aids	B20.*-B22.*, B24.*

fall_specific_01	Parkinson	G20
fall_specific_02	Gait disorders	R26.*
fall_specific_03	Polyneuropathies	G60.*-G65.*
fall_specific_04	Hip osteoarthritis	M16.*
fall_specific_05	Knee osteoarthritis	M17.*
fall_specific_06	Lumbar spinal stenosis	M48.06*
fall_specific_07	Bradycardia	R00.1
fall_specific_08	Hypersensitivity of the carotid sinus	G90.01
fall_specific_09	Urinary system disorders	N39.*
fall_specific_10	Blindness and impaired vision	H54.*
fall_specific_11	Vestibular function disorders	H81.*
fall_specific_12	Epilepsy	G40.*
fall_specific_13	Hearing loss	H90.*-H91.*
fall_specific_14	Osteoporosis	M80.*-M81.*
fall_specific_15	Osteoarthritis	M15.*-M19.*
fall_specific_16	Rheumatoid arthritis	M05.*-M06.*
fall_specific_17	Multiple sclerosis	G35.*
fall_specific_18	Gastric ulcer y duodenal	K25.*-K26.*
fall_specific_19	Ulcerative colitis	K51.*
fall_specific_20	Thyroid disorders	E00.*-E07.*
fall_specific_21	Persistent atrial fibrillation	I48.*
fall_specific_22	Asthma	J45.*
fall_specific_23	Insomnia	F51.0

Table SM3 - Hyperparameters and values for logistic regression.

**Hyperparameter**	**Explored values**
C	0.001, 0.01, 0.05, 0.1, 0.5, 1, 10, 50
penalty	*l1*, *l2*
solver	liblinear, *saga*
max_iter	100, 200, 500
class_weight	class_weights

Table SM4 - Hyperparameters and values for random forest.

**Hyperparameter**	**Explored values**
random_state	42
n_estimators	50, 100, 200, 300, 400
max_depth	5, 10, 15, 20, None
min_samples_split	2, 5, 10
min_samples_leaf	1, 2, 4
max_features	*auto*, *sqrt*, *log2*
criterion	*gini*, *entropy*
class_weight	class_weights

Table SM5 - Hyperparameters and values for extreme gradient boosting.

**Hyperparameter**	**Explored values**
objective	*binary_logistic*
random_state	42
n_estimators	50, 100, 200, 300, 500
max_depth	3, 5, 7, 9
learning_rate	0.01, 0.05, 0.1, 0.2
subsample	0.5, 0.75, 1
colsample_bytree	0.3, 0.5, 0.7
scale_pos_weight	scale_pos_weight

Table SM6 - Final list of variables included in the study.

**Source database**	**Variables**
Patient demographics and disease registry (PDDR) [n=48]	*sex, age, ses_group, deprivation_index, caregiver, fall, drugs_n, charlson_points, charlson_group, charlson_01, charlson_02, charlson_03, charlson_04, charlson_05, charlson_06, charlson_07, charlson_08, charlson_09, charlson_10, charlson_11, charlson_12, charlson_13, charlson_14, charlson_15, charlson_16, charlson_17, fall_specific_01, fall_specific_02, fall_specific_03, fall_specific_04, fall_specific_05, fall_specific_06, fall_specific_07, fall_specific_08, fall_specific_09, fall_specific_10, fall_specific_11, fall_specific_12, fall_specific_13, fall_specific_14, fall_specific_15, fall_specific_16, fall_specific_17, fall_specific_18, fall_specific_19, fall_specific_21, fall_specific_22, and fall_specific_23.*
Patient medication history (PMH) [n=177]	*A01A, A02A, A02B, A03A, A03B, A03F, A04A, A05A, A06A, A07A, A07B, A07C, A07D, A07E, A07F, A07X, A08A, A09A, A10A, A10B, A11A, A11C, A11D, A11G, A11H, A11J, A12A, A12B, A12C, A15Z, B01A, B02A, B02B, B03A, B03B, B05B, B05C, B05X, C01A, C01B, C01C, C01D, C01E, C02A, C02C, C02D, C03A, C03B, C03C, C03D, C03E, C04A, C05A, C05B, C05C, C07A, C07B, C07C, C07F, C08C, C08D, C09A, C09B, C09C, C09D, C09X, C10A, C10B, D01A, D01B, D02A, D03A, D03B, D05A, D05B, D06A, D06B, D07A, D07B, D07C, D07X, D08A, D10A, D10B, D11A, G01A, G02C, G03A, G03B, G03C, G03D, G03F, G03H, G03X, G04A, G04B, G04C, H01B, H01C, H02A, H03A, H03B, H03C, H04A, H05A, H05B, J01A, J01C, J01D, J01E, J01F, J01G, J01M, J01X, J02A, J04A, J05A, J07A, L01A, L01B, L01X, L02A, L02B, L04A, M01A, M01C, M02A, M03B, M04A, M05B, N01B, N02A, N02B, N02C, N03A, N04A, N04B, N05A, N05B, N05C, N06A, N06B, N06C, N06D, N07A, N07B, N07C, N07X, P01A, P01B, P02C, P03A, R01A, R02A, R03A, R03B, R03C, R03D, R05C, R05D, R06A, S01A, S01B, S01C, S01E, S01F, S01G, S01K, S01X, S02A, S02B, S02C, S02D, S03C, V03A, V04C, and V30Z.*
Healthcare utilization records (HUR) [n=6]	*visits_doctor_count_3_months, visits_nurse_count_3_months, out_patient_count_3_months, resources_emergency_count_3_months, hospitalization_count_3_months, and home_hospitalization_count_3_months.*

Table SM7 - Feature selection results.

Estimator	Variables selected	Nº variables
Logistic regression	*sex, age, caregiver, drugs_n, charlson_points, charlson_03, charlson_05, charlson_09, charlson_11, charlson_12, charlson_17, fall_specific_01, fall_specific_02, fall_specific_03, fall_specific_04, fall_specific_05, fall_specific_06, fall_specific_08, fall_specific_14, fall_specific_16, fall_specific_17, fall_specific_18, fall_specific_19, fall_specific_21, A01A, A02A, A02B, A03B, A03F, A04A, A05A, A06A, A07A, A07B, A07C, A07D, A07E, A07F, A07X, A08A, A09A, A10A, A10B, A11A, A11D, A11G, A11H, A11J, A12A, A12B, A12C, A15Z, B01A, B02A, B02B, B03B, B05B, B05C, B05X, C01A, C01B, C01C, C02A, C02C, C02D, C03A, C03B, C03C, C04A, C05A, C05B, C05C, C07B, C07C, C07F, C08C, C09A, C09C, C09D, C10A, C10B, D01A, D01B, D02A, D03A, D03B, D05A, D05B, D06A, D06B, D07A, D07B, D07X, D08A, D10A, D10B, D11A, G01A, G02C, G03A, G03B, G03C, G03D, G03F, G03H, G03X, G04A, G04B, G04C, H01B, H01C, H03B, H03C, H04A, H05A, H05B, J01A, J01C, J01D, J01E, J01F, J01G, J01M, J01X, J02A, J04A, J05A, L01A, L01B, L01X, L02A, L04A, M01A, M01C, M02A, M03B, M05B, N02A, N02B, N02C, N03A, N04B, N05A, N05B, N05C, N06A, N06B, N06C, N07A, N07B, N07C, N07X, P01A, P01B, P02C, P03A, R01A, R03A, R03B, R03C, R03D, R05C, R06A, S01A, S01B, S01C, S01E, S01F, S01K, S01X, S02A, S02B, S02D, S03C, V03A, V04C, V30Z, visits_doctor_count_3_months, visits_nurse_count_3_months, out_patient_count_3_months, resources_emergency_count_3_months, hospitalization_count_3_months, home_hospitalization_count_3_months*	183
Random forest	*sex, age, ses_group_2, deprivation_index, caregiver, drugs_n, charlson_points, charlson_group_2, charlson_01, charlson_02, charlson_03, charlson_04, charlson_05, charlson_06, charlson_07, charlson_08, charlson_09, charlson_10, charlson_11, charlson_12, charlson_13, charlson_14, charlson_15, charlson_16, charlson_17, fall_specific_01, fall_specific_02, fall_specific_03, fall_specific_04, fall_specific_05, fall_specific_06, fall_specific_07, fall_specific_08, fall_specific_09, fall_specific_10, fall_specific_11, fall_specific_12, fall_specific_13, fall_specific_14, fall_specific_15, fall_specific_16, fall_specific_17, fall_specific_18, fall_specific_19, fall_specific_21, fall_specific_22, fall_specific_23, A01A, A02A, A02B, A03A, A03B, A03F, A04A, A05A, A06A, A07A, A07C, A07D, A07E, A07F, A08A, A09A, A10A, A10B, A11C, A11D, A11G, A11H, A11J, A12A, A12B, A12C, B01A, B02A, B02B, B03A, B03B, B05B, C01A, C01B, C01C, C01D, C01E, C02A, C02C, C02D, C03A, C03B, C03C, C03D, C03E, C04A, C05A, C05B, C05C, C07A, C07B, C07C, C07F, C08C, C08D,*	209

Estimator	Variables selected	Nº variables
	C09A, C09B, C09C, C09D, C09X, C10A, C10B, D01A, D02A, D03A, D05A, D05B, D06A, D06B, D07A, D07C, D07X, D10A, D10B, D11A, G01A, G02C, G03B, G03C, G03D, G03H, G03X, G04A, G04B, G04C, H01B, H01C, H02A, H03A, H03B, H04A, H05A, H05B, J01A, J01C, J01D, J01E, J01F, J01M, J01X, J02A, J04A, J05A, L01A, L01B, L01X, L02A, L02B, L04A, M01A, M01C, M02A, M03B, M04A, M05B, N01B, N02A, N02B, N02C, N03A, N04A, N04B, N05A, N05B, N05C, N06A, N06B, N06C, N06D, N07A, N07B, N07C, P01B, R01A, R03A, R03B, R03C, R03D, R05C, R05D, R06A, S01A, S01B, S01C, S01E, S01F, S01G, S01K, S01X, S02A, S02B, S02C, S03C, V03A, V04C, V30Z, visits_doctor_count_3_months, visits_nurse_count_3_months, out_patient_count_3_months, resources_emergency_count_3_months, hospitalization_count_3_months, home_hospitalization_count_3_months	
Extreme gradient boosting	*sex, age, ses_group_2, deprivation_index, caregiver, drugs_n, charlson_points, charlson_01, charlson_02, charlson_03, charlson_04, charlson_05, charlson_06, charlson_07, charlson_08, charlson_09, charlson_10, charlson_11, charlson_12, charlson_13, charlson_14, charlson_15, charlson_16, charlson_17, fall_specific_01, fall_specific_02, fall_specific_03, fall_specific_04, fall_specific_05, fall_specific_06, fall_specific_07, fall_specific_09, fall_specific_10, fall_specific_11, fall_specific_12, fall_specific_13, fall_specific_14, fall_specific_15, fall_specific_16, fall_specific_18, fall_specific_19, fall_specific_21, fall_specific_22, fall_specific_23, A02A, A02B, A03A, A03F, A05A, A06A, A07A, A07D, A07F, A08A, A09A, A10A, A10B, A11C, A11D, A11H, A12A, A12B, A12C, B01A, B03A, B03B, B05B, C01A, C01B, C01D, C01E, C02A, C02C, C03A, C03B, C03C, C03D, C03E, C04A, C05A, C05B, C05C, C07A, C07B, C07F, C08C, C08D, C09A, C09B, C09C, C09D, C10A, C10B, D01A, D03A, D05A, D05B, D06A, D07A, D07X, D10A, D10B, D11A, G01A, G02C, G03B, G03C, G04B, G04C, H02A, H03A, H03B, H04A, H05A, H05B, J01C, J01D, J01E, J01F, J01X, J05A, L02A, L04A, M01A, M02A, M03B, M04A, M05B, N01B, N02A, N02B, N03A, N04A, N04B, N05A, N05B, N05C, N06A, N06D, N07A, N07C, R01A, R03A, R03B, R03D, R05C, R05D, R06A, S01A, S01B, S01C, S01E, S01F, S01K, S01X, S03C, V03A, V30Z, visits_doctor_count_3_months, visits_nurse_count_3_months, out_patient_count_3_months, resources_emergency_count_3_months, hospitalization_count_3_months, home_hospitalization_count_3_months*	164

Table SM8 - Hyperparameter optimization results.

**Feature selection estimator**	**Algorithm trained**	**Best parameters**
Logistic regression (n=183)	Logistic regression	*C: 0.5, max_iter: 200, penalty: l1, solver: liblinear*
	Random forest	*criterion: entropy, max_depth: none, max_features: log2, min_samples_leaf: 1, min_samples_split: 5, n_estimators: 400*
	Extreme gradient boosting	*colsample_bytree: 0.7, learning_rate: 0.05, max_depth: 3, n_estimators: 300, subsample: 0.5*
Random forest (n=209)	Logistic regression	*C: 0.1, max_iter: 200, penalty: l1, solver: liblinear*
	Random forest	*criterion: entropy, max_depth: none, max_features: auto, min_samples_leaf: 2, min_samples_split: 10, n_estimators: 400*
	Extreme gradient boosting	*colsample_bytree: 0.3, learning_rate: 0.05, max_depth: 3, n_estimators: 300, subsample: 0.75*
Extreme gradient boosting (n=164)	Logistic regression	*C: 0.5, max_iter: 100, penalty: l1, solver: liblinear*
	Random forest	*criterion: entropy, max_depth: none, max_features: auto, min_samples_leaf: 4, min_samples_split: 2, n_estimators: 400*
	Extreme gradient boosting	*colsample_bytree: 0.7, learning_rate: 0.1, max_depth: 3, n_estimators: 200, subsample: 0.75*

Table SM9 - Evaluation results at the default threshold.

Feature selection estimator	Algorithm trained	AUCROC	AUCPR	Accuracy	Recall	Precision	True positive	False positive	True negative	False negative
Logistic regression	Logistic regression	0.706	0.225	0.675	0.596	0.182	443	1,985	4,312	300
	Random forest	0.708	0.217	0.894	0.000	0.000	0	0	6,297	743
	Extreme gradient boosting	0.711	0.224	0.670	0.622	0.184	462	2,045	4,252	281
Random forest	Logistic regression	0.706	0.224	0.670	0.590	0.179	428	2,015	4,282	305
	Random forest	0.714	0.218	0.894	0.017	0.406	13	19	6,278	730
	Extreme gradient boosting	0.712	0.225	0.666	0.616	0.181	458	2,069	4,228	285
Extreme gradient boosting	Logistic regression	0.706	0.225	0.675	0.596	0.182	443	1,989	4,308	300
	Random forest	0.714	0.217	0.887	0.060	0.319	44	94	6,203	699
	Extreme gradient boosting	0.709	0.217	0.677	0.608	0.186	452	1,982	4,315	291

Figure SM 1 - Shapley additive explanations values (SHAP) of the 10 most influential logistic regression-selected features by classification algorithm.

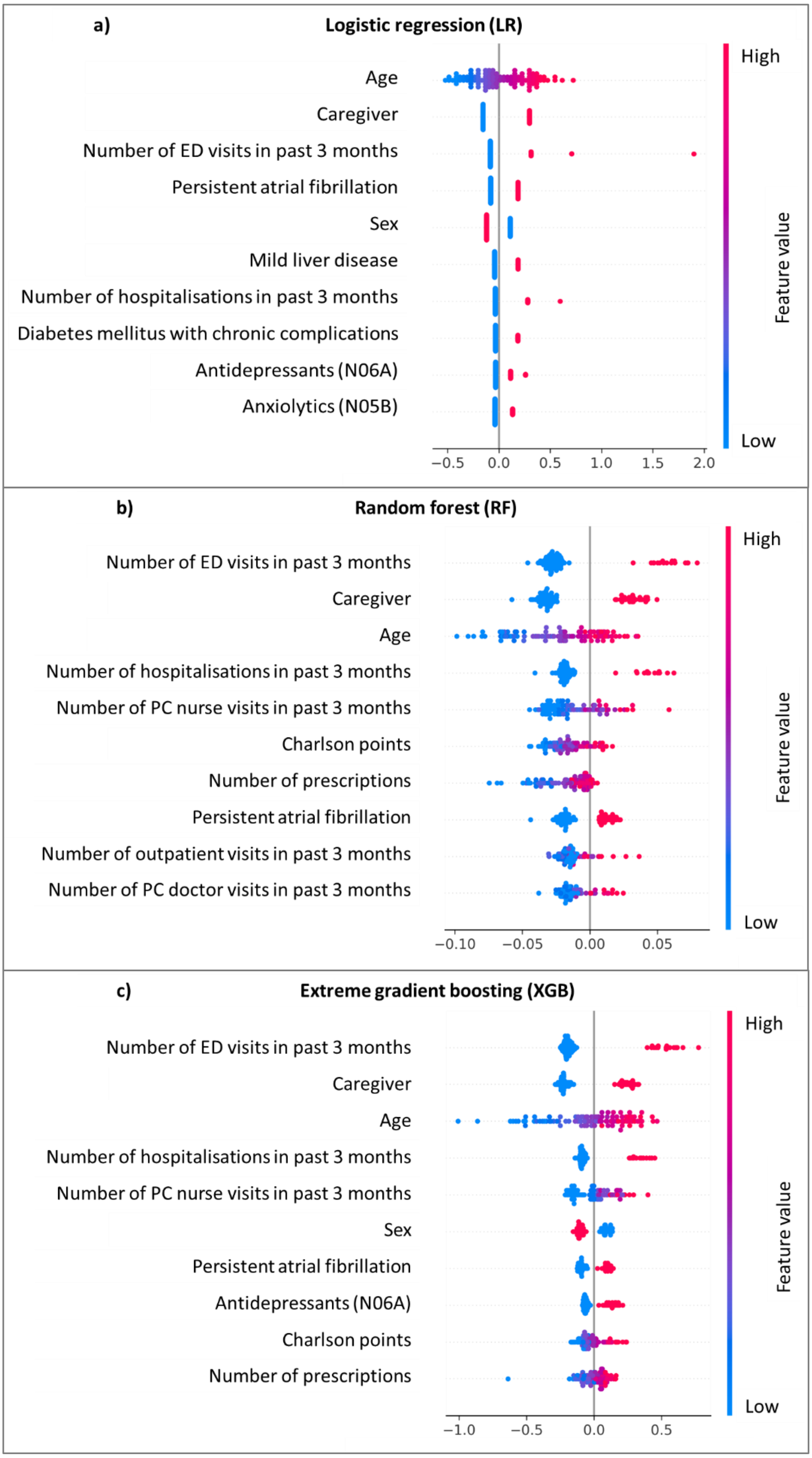

Figure SM 2 - Shapley additive explanations values (SHAP) of the 10 most influential random forest-selected features by classification algorithm.

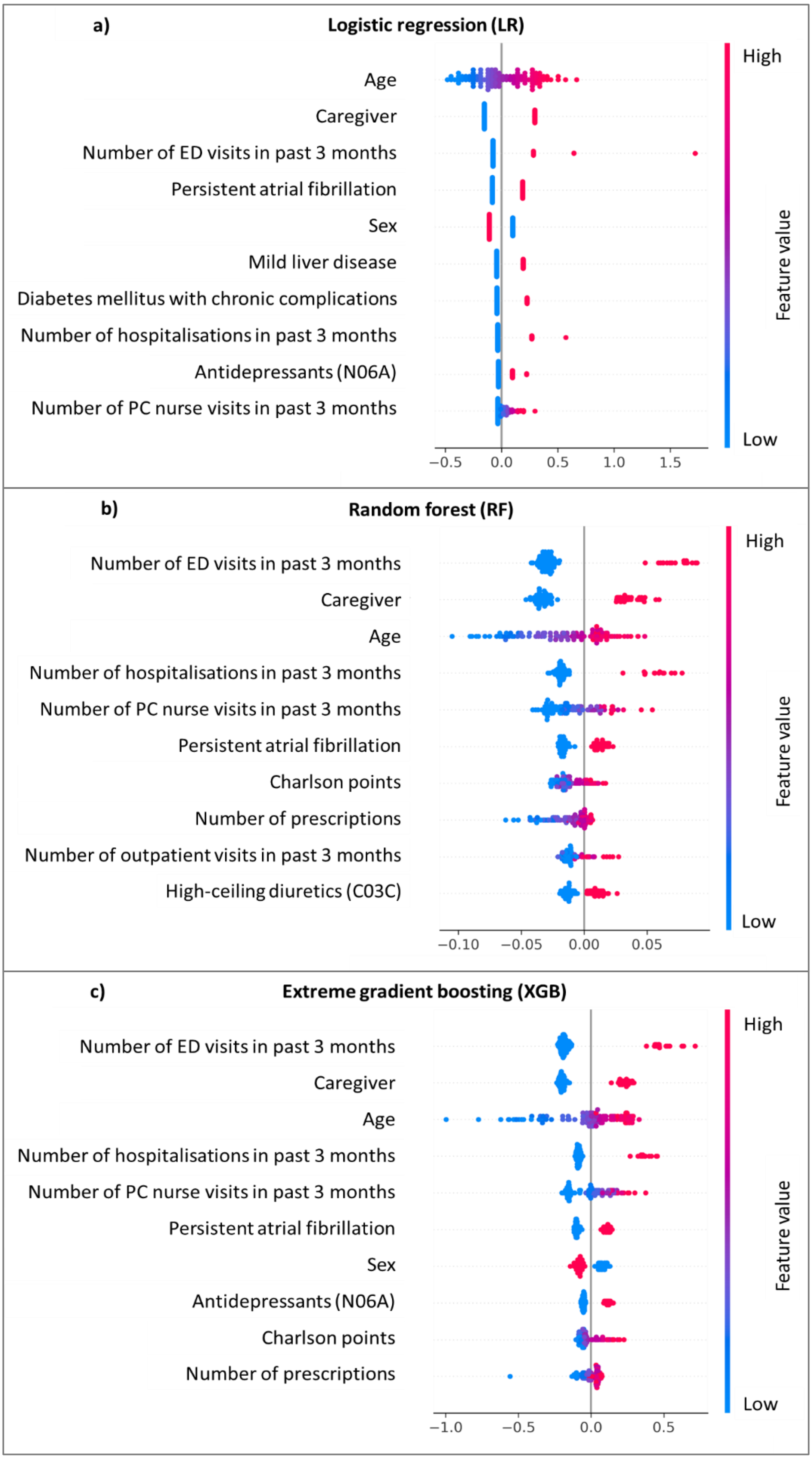

Figure SM 3 - Shapley additive explanations values (SHAP) of the 10 most influential extreme gradient boosting-selected features by classification algorithm.

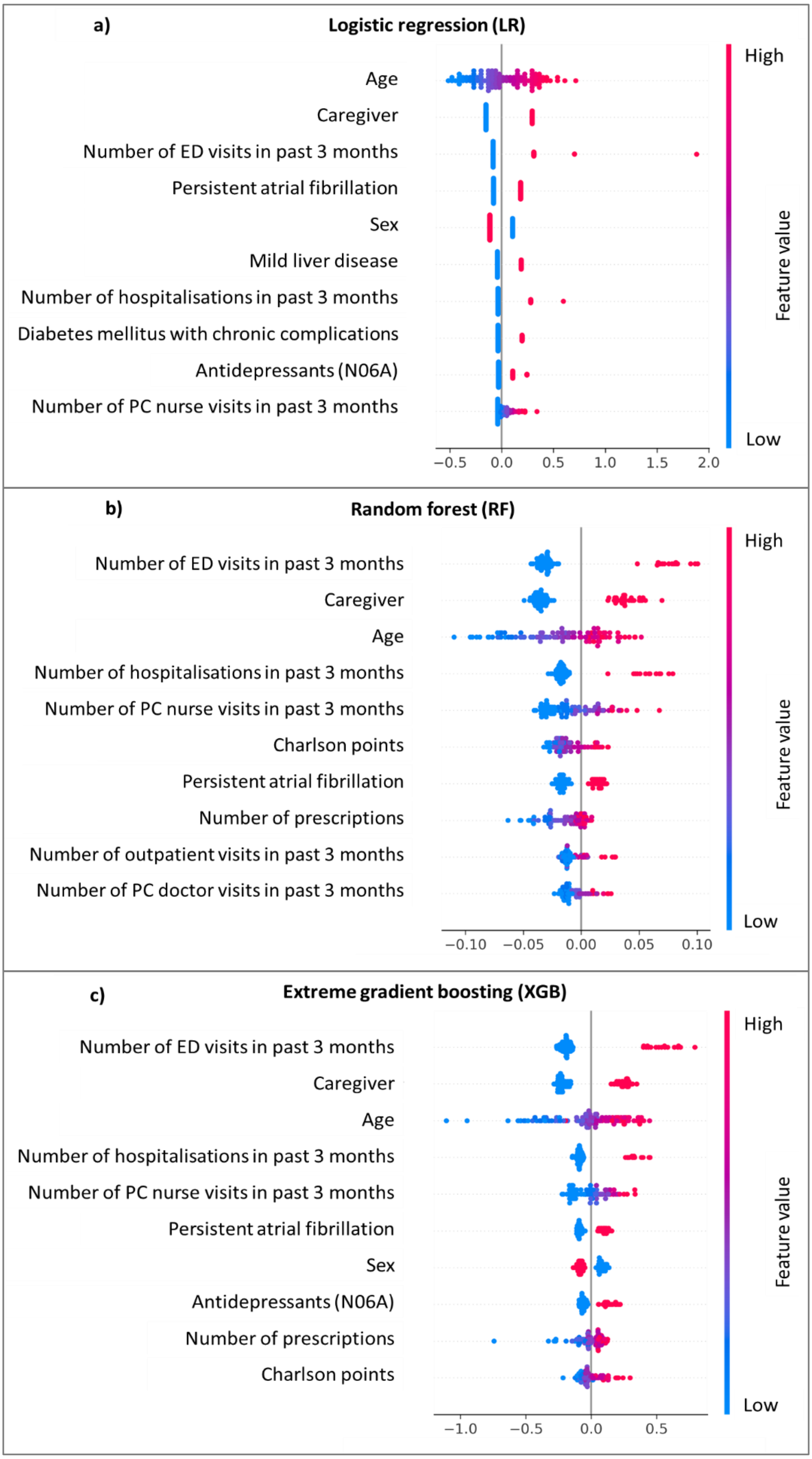

Supplement: Supplementary file 1 [file Data_Sheet_1.PDF]
